# Supplementary material for: Splice-Junction-Based Mapping of Alternative Isoforms in the Human Proteome
Source: Cell Rep. Author manuscript; Available in PMC 2020 Jan 15. (PMC6961840; doi:10.1016/j.celrep.2019.11.026)
Supplement: 3 [file NIHMS1546469-supplement-3.zip › DF2/PXD0010154/Esophagus-23-Q9Y2J2-TDTAADGETTATEELEK.pdf]

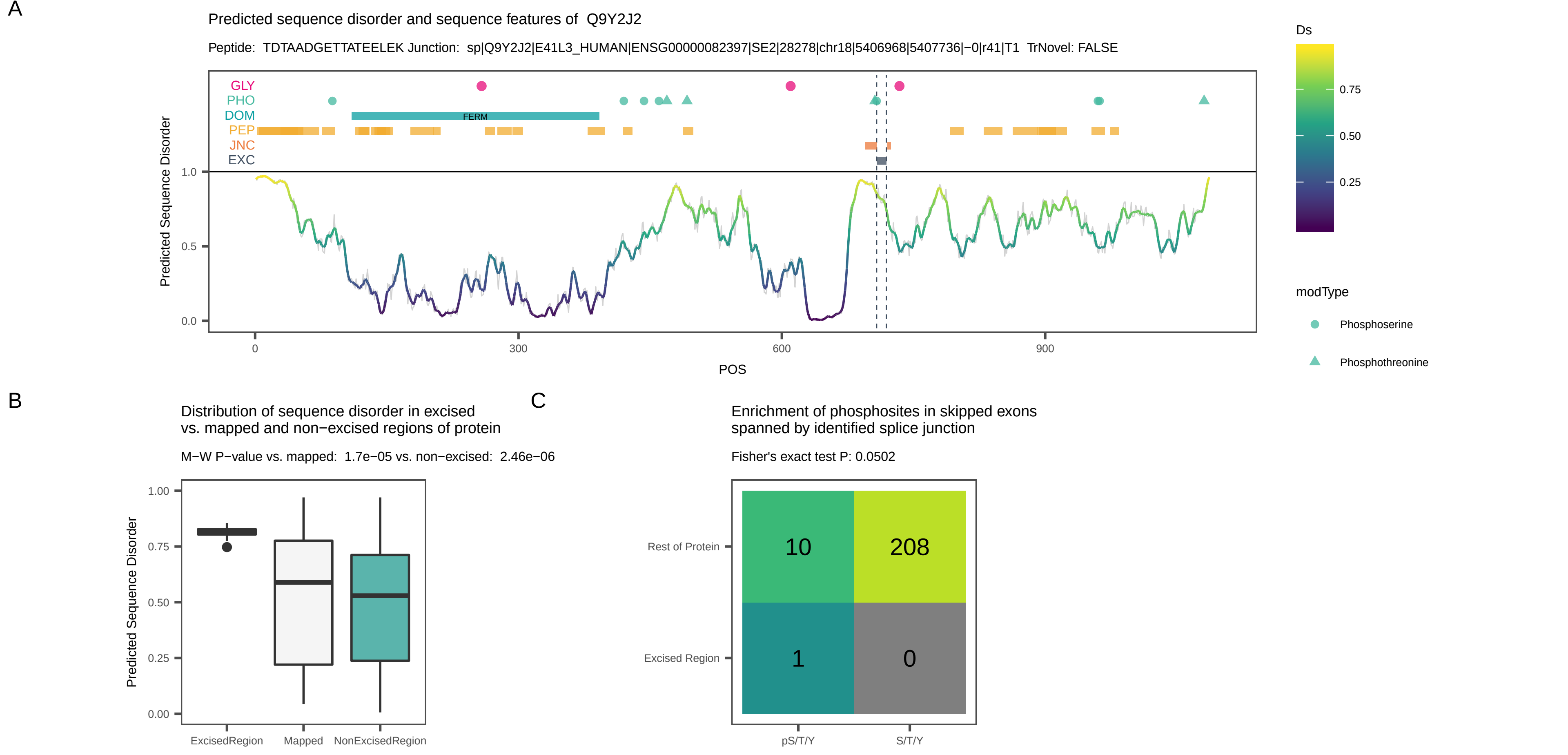
A
Predicted sequence disorder and sequence features of Q9Y2J2
Peptide: TDTAADGETTATEELEK Junction: sp|Q9Y2J2|E41L3_HUMAN|ENSG00000082397|SE2|28278|chr18|5406968|5407736|−0|r41|T1 TrNovel: FALSE
GLY
PHO
DOM
PEP
JNC
EXC
FERM
Predicted Sequence Disorder
1.0
0.5
0.0
0
300
600
900
POS
Ds
0.75
0.50
0.25
modType
Phosphoserine
Phosphothreonine
B
Distribution of sequence disorder in excised
vs. mapped and non−excised regions of protein
M−W P−value vs. mapped: 1.7e−05 vs. non−excised: 2.46e−06
Predicted Sequence Disorder
1.00
0.75
0.50
0.25
0.00
ExcisedRegion
Mapped
NonExcisedRegion
C
Enrichment of phosphosites in skipped exons
spanned by identified splice junction
Fisher's exact test P: 0.0502
Rest of Protein
Excised Region
10
208
1
0
pS/T/Y
S/T/Y
